# Supplementary material for: Loss of Mpdz impairs ependymal cell integrity leading to perinatal‐onset hydrocephalus in mice
Source: EMBO Mol Med. 2017 May 12;9(7):890–905. doi: 10.15252/emmm.201606430 (PMC5494508; doi:10.15252/emmm.201606430)
Supplement: Supplementary file 1 — Appendix [file EMMM-9-890-s001.pdf]

Appendix

Loss of Mpdz impairs ependymal cell integrity leading to perinatal-onset hydrocephalus in mice

Anja Feldner, M. Gordian Adam, Fabian Tetzlaff, Iris Moll, Dorde Komljenovic, Felix Sahm, Tobias Bäuerle, Hiroshi Ishikawa, Horst Schroten, Thomas Korff, Ilse Hofmann, Hartwig Wolburg, Andreas von Deimling, Andreas Fischer

Table of Contents

Appendix Figure S1.

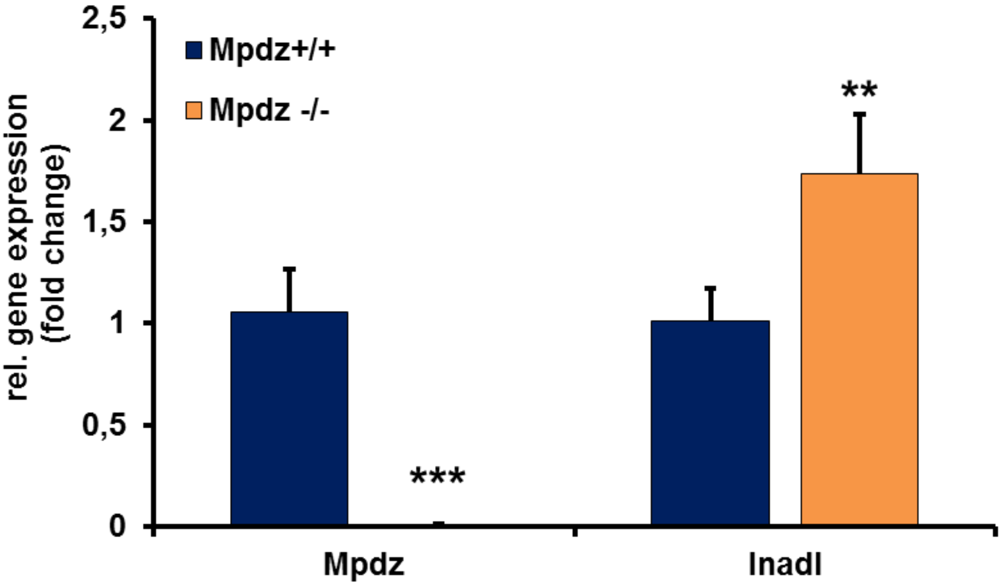

Appendix Figure S1. Higher *Inadl* mRNA expression after loss of *Mpdz*.
Lung endothelial cells were freshly isolated from *Mpdz*$^{-/-}$ and littermate *Mpdz*$^{+/+}$ mice at postnatal day 7. Expression of relative *Mpdz* and *Inadl* mRNA levels were determined by real-time PCR. n = 3 animals per genotype. **, P=0.006; ***,P=0.000076. Data are presented as mean ± SD, (two-sided, unpaired Student's t-test).
