# Supplementary material for: Loss of Mpdz impairs ependymal cell integrity leading to perinatal‐onset hydrocephalus in mice
Source: EMBO Mol Med. 2017 May 12;9(7):890–905. doi: 10.15252/emmm.201606430 (PMC5494508; doi:10.15252/emmm.201606430)
Supplement: Supplementary file 2 — Expanded View Figures PDF [file EMMM-9-890-s002.pdf]

Expanded View Figures

Figure EV1. Loss of *Mpdz* does not alter the brain microvasculature and endothelial-restricted loss of *Mpdz* does not cause hydrocephalus.

A Brain sections from $Mpdz^{-/-}$ and littermate $Mpdz^{+/+}$ mice were stained for the endothelial marker CD31. Scale bars, 50 μm. Graph showing relative changes in microvessel density. n = 3 animals per genotype; n.s., not significant (unpaired two-sided Student's t-test). Data are presented as mean ± SD.

B Brain section stained for the tight junction proteins claudin-5 and ZO-1. No differences were observed between $Mpdz^{-/-}$ and littermate $Mpdz^{+/+}$ mice at P7. Scale bar, 50 μm.

C Transmission electron micrograph of cortical brain capillaries shows no difference in tight junction assembly between $Mpdz^{-/-}$ and $Mpdz^{+/+}$ littermates. Boxes are highlighting endothelial cell junctions. One representative box shows the area as a zoom-in. Scale bars, 1 μm and 100 nm. EC, endothelial cell; PC, pericyte.

D Western blot to determine Mpdz protein expression in isolated lung endothelial cells from adult Tie2-Cre;$Mpdz^{\Delta EC/\Delta EC}$ mice.

E Endothelial cell-specific *Mpdz*-deficient mice (ΔEC/ΔEC) developed normally with no indication for hydrocephalus (n > 200 ΔEC/ΔEC observed for more than four months of age). H&E staining of brain sections from three-month-old mice.

Source data are available online for this figure.

© 2017 The Authors

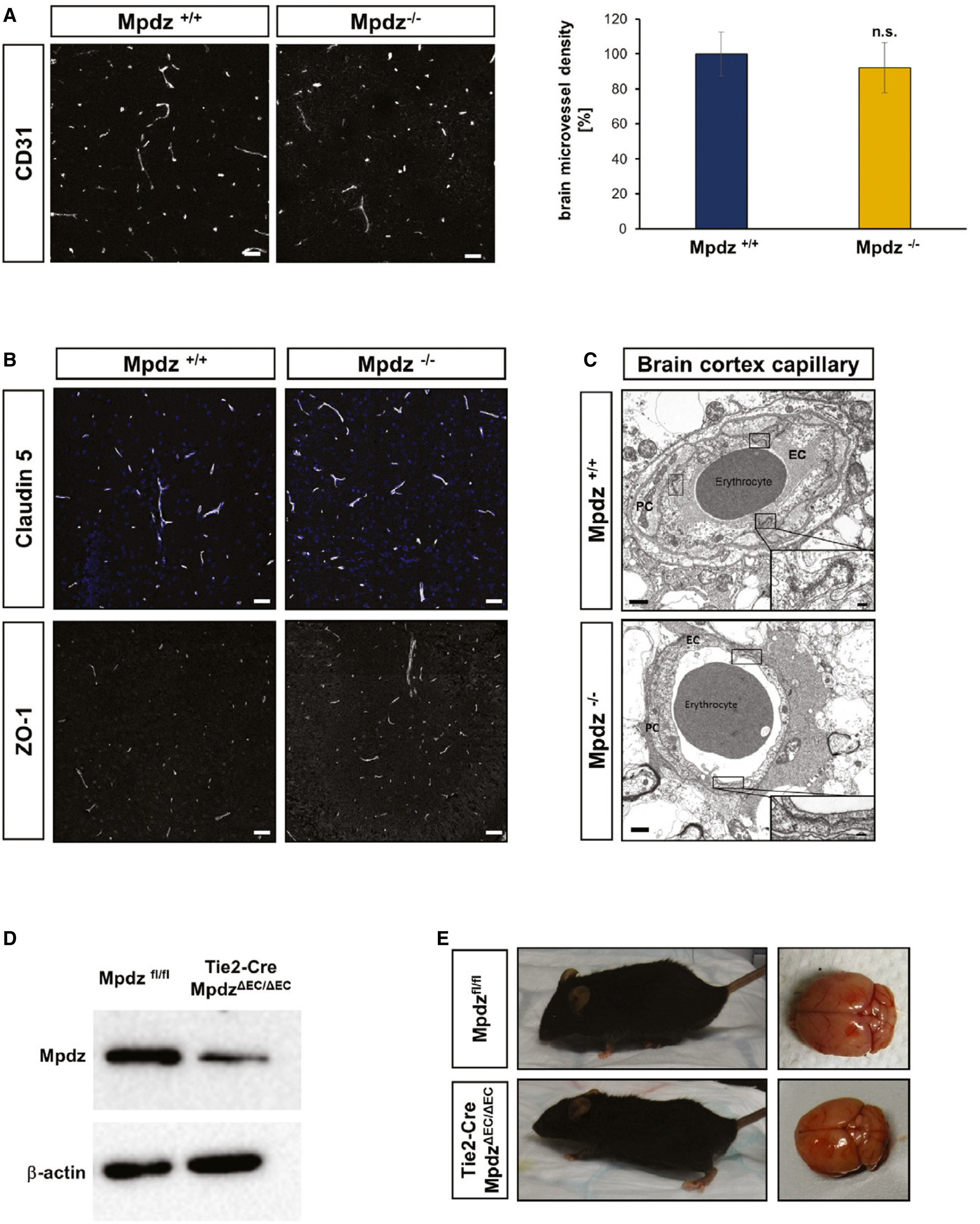
A
Mpdz +/+
Mpdz -/-
CD31
brain microvessel density [%]
120
100
80
60
40
20
0
n.s.
Mpdz +/+
Mpdz -/-
B
Mpdz +/+
Mpdz -/-
Claudin 5
ZO-1
C
Brain cortex capillary
Mpdz +/+
Mpdz -/-
EC
Erythrocyte
PC
D
Mpdz fl/fl
Tie2-Cre MpdzΔEC/ΔEC
Mpdz
β-actin
E
Mpdzfl/fl
Tie2-Cre MpdzΔEC/ΔEC

Figure EV1.

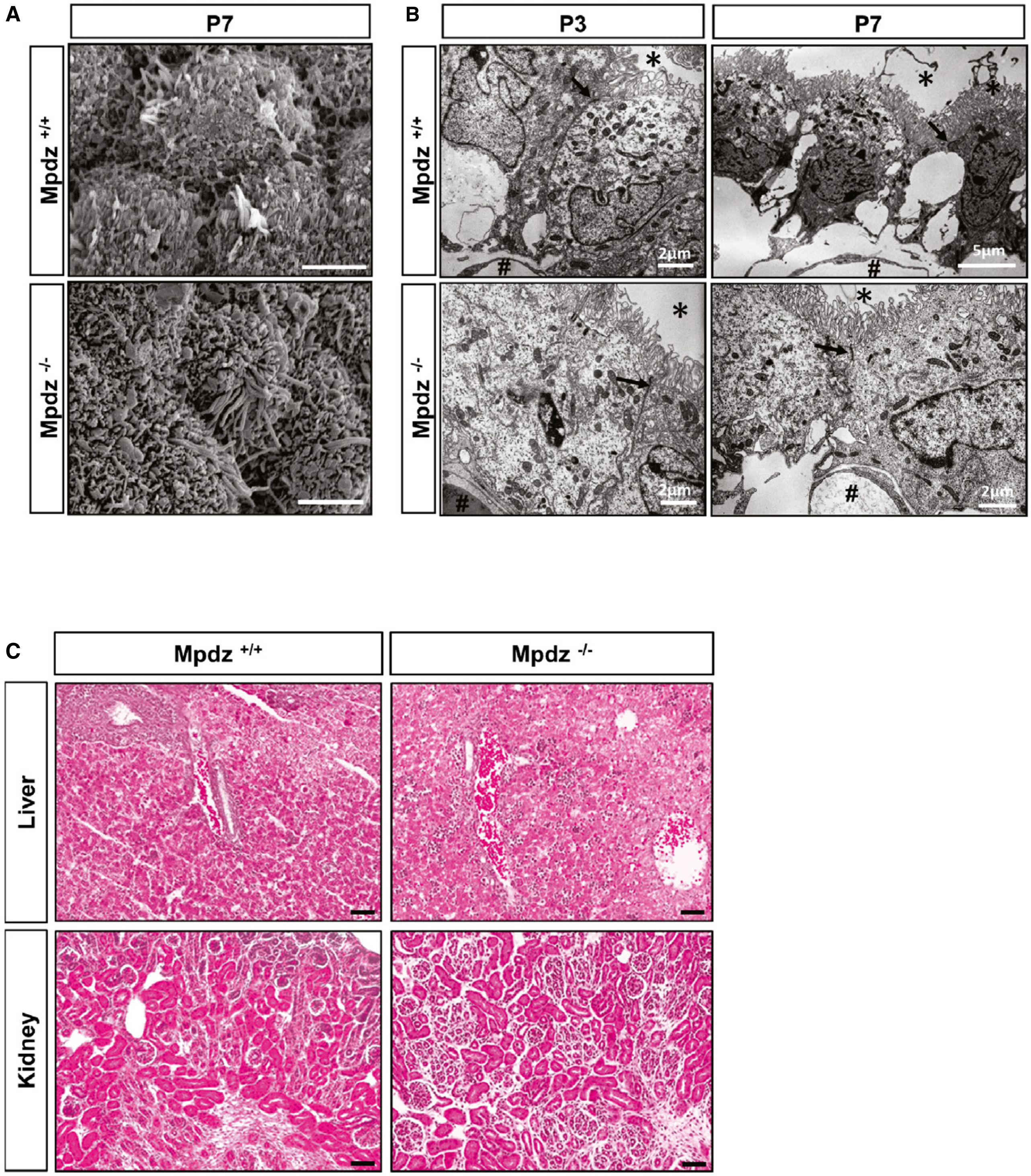

Figure EV2. Normal morphology of choroid plexus, liver, and kidney in *Mpdz*$^{-/-}$ mice.

A Scanning electron micrograph of choroid plexus epithelium of *Mpdz*$^{-/-}$ and *Mpdz*$^{+/+}$ littermate mice at P7 shows no morphological abnormalities in cilia or microvilli. Scale bars, 3 µm.

B Electron microscopic analysis of the choroid plexus from a lateral ventricle of *Mpdz*$^{-/-}$ and *Mpdz*$^{+/+}$ littermates at postnatal day 3 and day 7. The plexus epithelial cells are typically polarized, carry apical microvilli (asterisks), and are interconnected by tight junctions (arrows). In the stroma, beneath the epithelium the fenestrated capillaries are seen (hashtags; for comparison, see Wolburg & Paulus, 2010). Scale bars indicated in images.

C Kidney and liver of *Mpdz*$^{-/-}$ and *Mpdz*$^{+/+}$ littermates at P7 were embedded in paraffin. H&E staining of sections shows no overt differences between the genotypes. Scale bar, 50 µm.

© 2017 The Authors

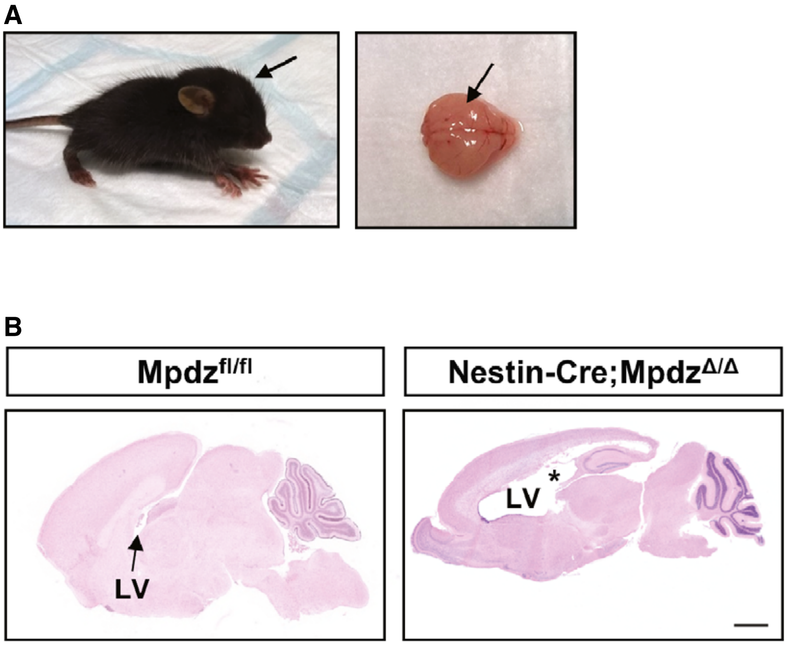

Figure EV3. *Mpdz* inactivation using Nestin-Cre mice causes hydrocephalus.

Floxed *Mpdz* mice were crossed with Nestin-Cre to delete *Mpdz* in glial and neuronal precursors.

A Dome-shaped skull (arrow) and the enlarged brain hemispheres (arrow) at postnatal day 7.

B Lateral ventricles (LV) were enlarged (asterisk). Scale bar, 1,000 μm.

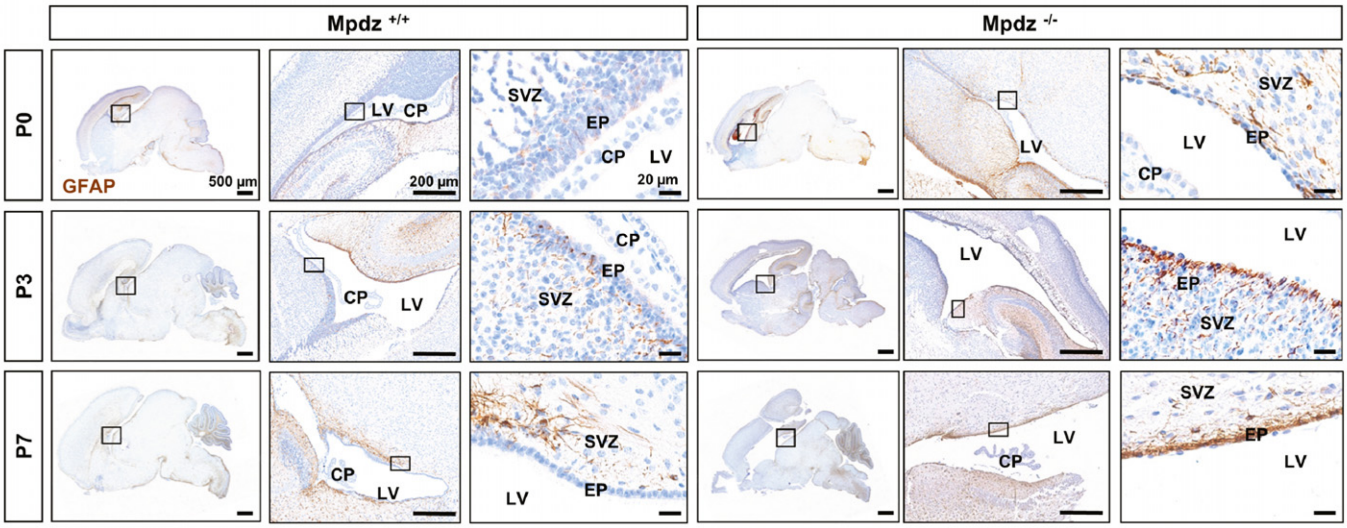

Figure EV4. Reactive astrogliosis in *Mpdz*-deficient mice.

Staining of glial fibrillary acidic protein (GFAP, DAB stain: brown color) at postnatal days 0, 3, and 7 (P0, P3, and P7) on paraffin-embedded brain sections. GFAP expression is more intense and widespread in *Mpdz*-deficient mice compared to wild-type littermate controls. Boxed areas are shown in higher magnification at right row. Please note that the *Mpdz*$^{-/-}$ brain section (P3) is derived from the same specimen than in Fig 3. SVZ, subventricular zone; CP, choroid plexus; EP, ependymal cells. Scale bars: 500 μm left row, 200 μm middle row, 20 μm right row.

 © 2017 The Authors
