# Supplementary figures and images for: Loss of Mpdz impairs ependymal cell integrity leading to perinatal‐onset hydrocephalus in mice
Source: EMBO Mol Med. 2017 May 12;9(7):890–905. doi: 10.15252/emmm.201606430 (PMC5494508; doi:10.15252/emmm.201606430)

Figure EV1 D

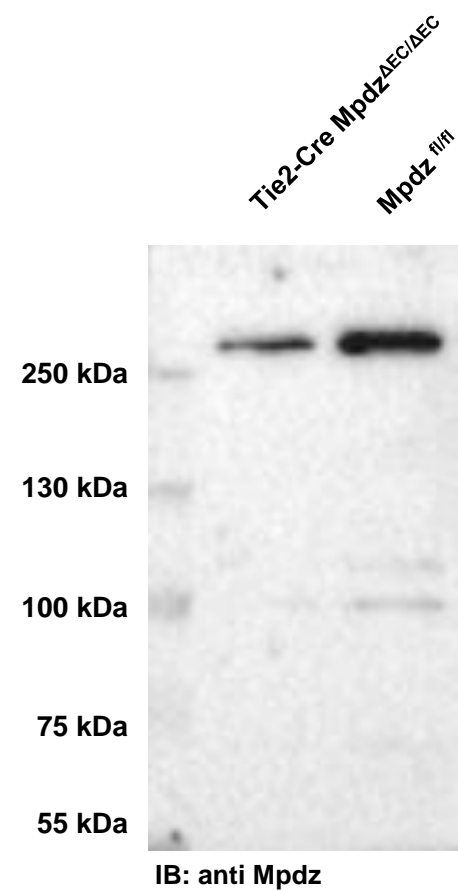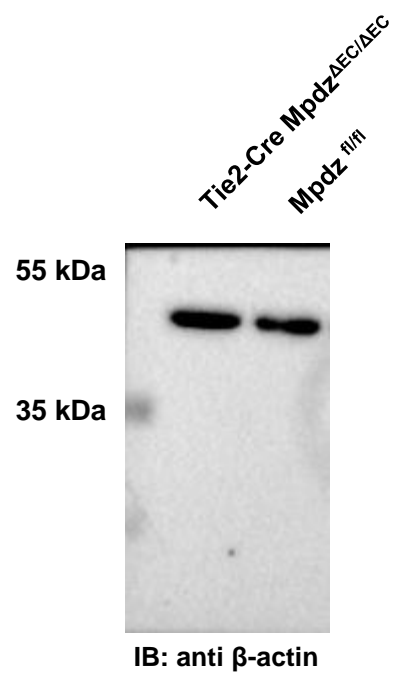

Supplement: Supplementary file 3 — Source Data for Expanded View [file EMMM-9-890-s005.zip › Source_data_EV_Figs/Source_data_Figure_EV1.pdf]

Figure 1 B

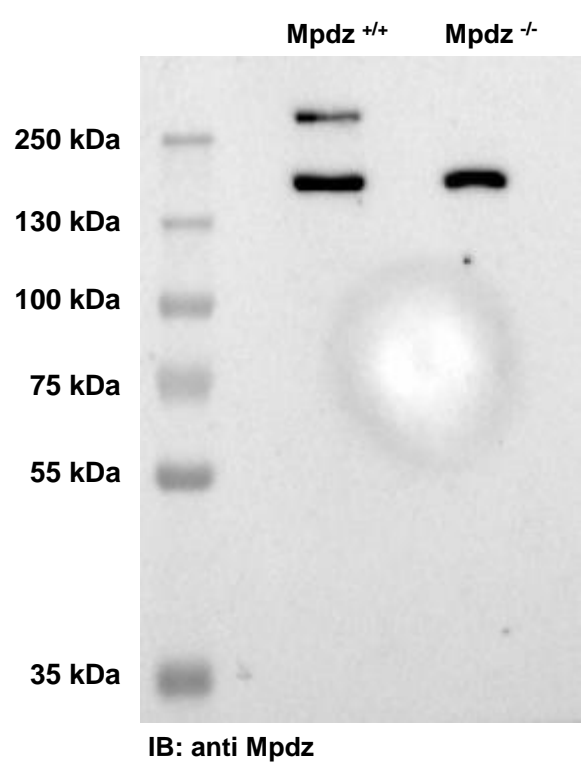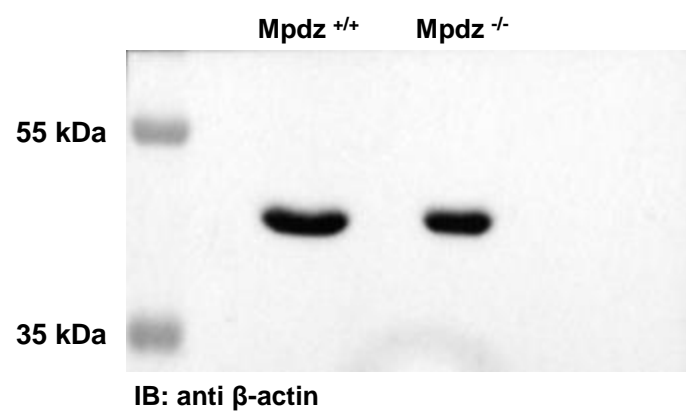

Figure 1 D

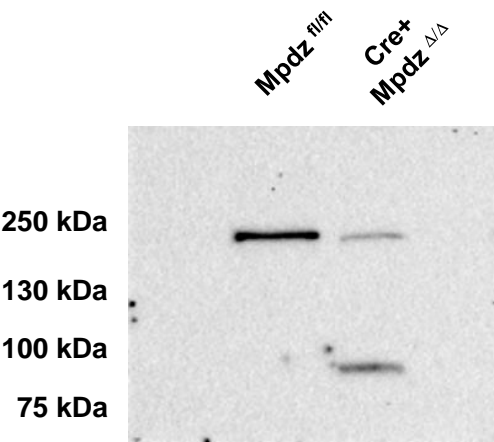

IB: anti Mpdz

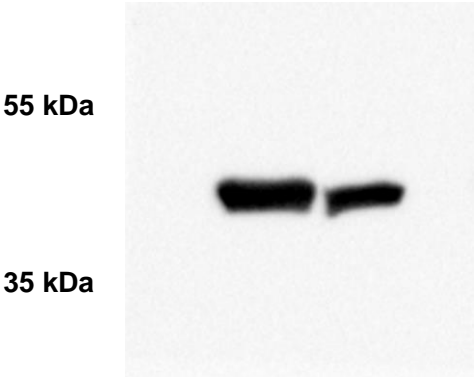

IB: anti β-actin

Supplement: Supplementary file 5 — Source Data for Figure 1 [file EMMM-9-890-s003.pdf]
