# Supplementary material for: Loss of Mpdz impairs ependymal cell integrity leading to perinatal‐onset hydrocephalus in mice
Source: EMBO Mol Med. 2017 May 12;9(7):890–905. doi: 10.15252/emmm.201606430 (PMC5494508; doi:10.15252/emmm.201606430)
Supplement: Supplementary file 6 — Source Data for Figure 6 [file EMMM-9-890-s004.pdf]

Figure 6 C

	72h	72h [%]	Average	SD
si-control	24,817	100	100	0
	20,867	100		
	38,119	100		
	48,505	100		
	26,497	100		
si-MPDZ	8,996	36,24935	54,53284	12,89514
	12,24	58,65721		
	27,237	71,45256		
	24,11	49,70622		
	14,997	56,59886		

	72h	72h [%]	Average	SD
sh-control	50,758	100	100	0
	71,567	100		
	27,36	100		
sh-MPDZ	15,674	30,87986	40,55056	23,15883
	17,029	23,79449		
	18,325	66,97734		

Figure 6 E

	RhoA	GAPDH	RhoA/Gapdh	active RhoA	active RhoA/total RhoA				
WT-1	2.707.972	5.180.344	0,523	0,7525	1,440				
KO-1	1.883.277	4.752.869	0,396	1,0875	2,745			mean	SD
WT-2	4.745.571	9.353.382	0,507	0,467	0,920		Mpdz +/+	1,191	0,26028954
KO-2	1.246.451	4.721.354	0,264	0,8135	3,081		Mpdz -/-	2,602	0,56451339
WT-3	657.092	848.243	0,775	0,9405	1,214				
KO-3	257.728	591.920	0,435	0,862	1,980				
